# Generalized bullous fixed-drug eruption secondary to the influenza vaccine

**DOI:** 10.1016/j.jdcr.2018.07.013

**Published:** 2018-10-12

**Authors:** Rachel Chikowski Byrd, Kimberly J. Mournighan, Michael Baca-Atlas, Margaret R. Helton, Natalie Z. Sun, Marni B. Siegel

**Affiliations:** a Department of Dermatology, University of North Carolina at Chapel Hill, Chapel Hill, North Carolina; c Department of Family Medicine, University of North Carolina at Chapel Hill, Chapel Hill, North Carolina; b University of North Carolina School of Medicine, University of North Carolina at Chapel Hill, Chapel Hill, North Carolina; d Lineberger Comprehensive Cancer Center, University of North Carolina at Chapel Hill, Chapel Hill, North Carolina

**Keywords:** drug, fixed drug eruption, generalized bullous, EM, erythema multiforme, FDE, fixed drug eruption, GBFDE, generalized bullous fixed drug eruption, SJS, Stevens-Johnson syndrome, TEN, toxic epidermal necrolysis

## Introduction

Fixed drug eruption (FDE) is a type IV hypersensitivity reaction characterized by recurrence of lesions at identical sites with each exposure to the offending medication. After morbilliform exanthems, FDE is the most common cutaneous drug reaction.^1^ Generalized bullous fixed drug eruption (GBFDE) is a variant of FDE that can present rarely with significant, life-threatening body surface involvement akin to Stevens-Johnson syndrome (SJS) and toxic epidermal necrolysis (TEN).^2^ There are few reported cases of GBFDE occurring after the influenza vaccine, and all occurred after decades of receiving the vaccine.^3^, ^4^ Here we report a rare case of GBFDE occurring as a result of administration of the influenza vaccine.

## Case report

A 67-year-old African-American woman with type 2 diabetes mellitus, hypertension, chronic obstructive pulmonary disease, and coronary artery disease presented with an exquisitely painful bullous eruption.

**Fig 1:**
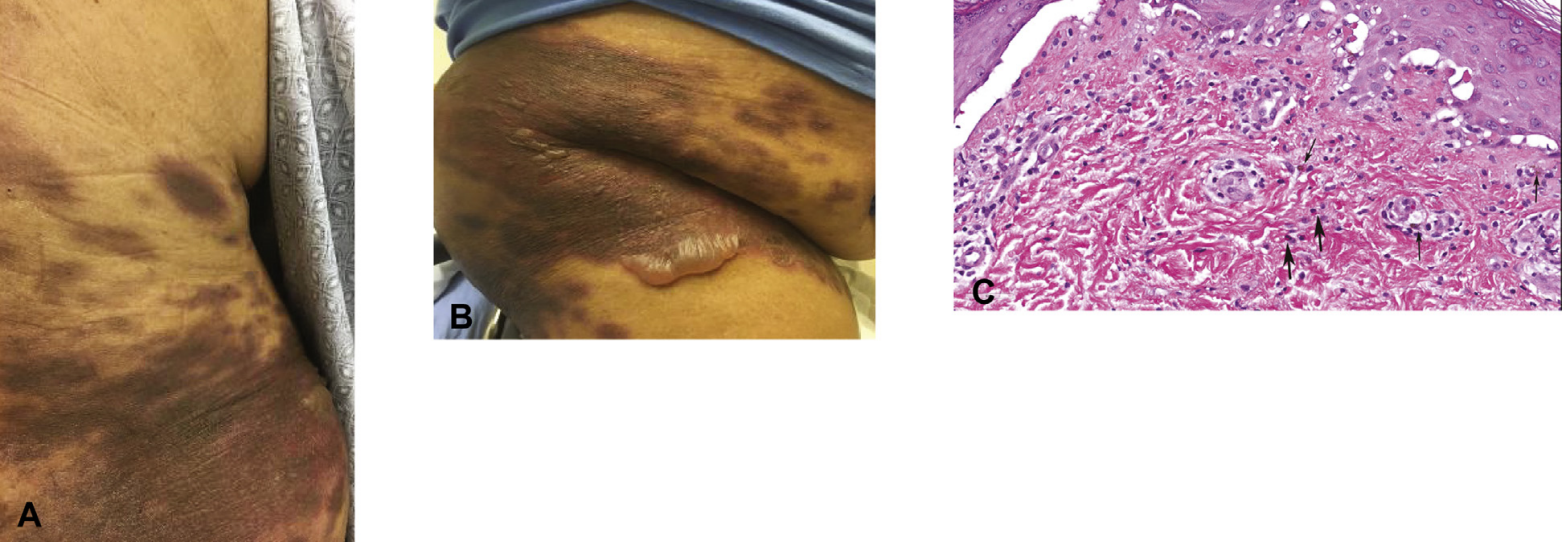
Generalized bullous fixed drug eruption after the influenza vaccine. **A**, Well-demarcated red-brown patches with surrounding rim of erythema in a background of postinflammatory hyperpigmentation. **B**, Numerous coalescing bullae. **C**, Vacuolar interface dermatitis, dyskeratotic keratinocytes with a lympho-eosinophilic infiltrate (*arrows*). (**C**, Hematoxylin- eosin stain; original magnification: ×40.)

Three days after administration of the quadrivalent influenza vaccine, she presented with multiple 2.5- to 5-cm hyperpigmented patches and plaques with a peripheral rim of erythema (Fig 1, *A*), some with overlying flaccid bullae, located on her bilateral hips and lower back (Fig 1, *B*). She had a background of numerous hyperpigmented patches coalescing on the back, flanks, and buttocks consistent with postinflammatory hyperpigmentation as a result of a similar bullous eruption after the influenza vaccine 1 year prior.

A thorough medication review failed to find any other recent medication changes. New bullae continued to appear across her groin, lower back, buttocks, axillae, and abdomen over the subsequent days. Although a few oral erosions involving the hard palate and vermilion lips were present, there was no evidence of ocular or genital mucosal involvement. There was complete sparing of the distal extremities including the palms and soles. She remained afebrile with a normal complete blood count and comprehensive metabolic panel.

Biopsy found vacuolar interface dermatitis with subepidermal vesicle formation, epidermal necrosis, marked pigment incontinence, and mild lympho-eosinophilic infiltrate (Fig 1, *C*). These findings were consistent with the biopsy results from 1 year prior. The episode at that time was of a similar clinical presentation and resolved after 7 days of topical clobetasol 0.05% ointment.

During the second bullous eruption, the patient required use of both topical clobetasol ointment and an oral prednisone taper. Within 2 days of hospitalization, new lesions continued to appear, now covering 15% of her total body surface area. The patient was transferred to the burn intensive care unit for aggressive wound care. She was discharged after 14 days in the burn intensive care unit, with complete resolution at 30 days.

## Discussion

FDE is characterized by discrete, well-demarcated, violaceous, circular patches or plaques that are typically self-limited. However, a life-threatening form of FDE with numerous coalescing flaccid bullae can develop with repeated exposures.^1^ Our patient's second influenza vaccination essentially served as a systemic provocation test, which was positive. Here, we present a unique case of GBFDE secondary to the influenza vaccine.

Taken together, our patient's reproducible bullous eruption and supportive biopsy results after receiving the influenza vaccine were consistent with GBFDE. There are multiple clinical factors that favor GBFDE over erythema multiforme (EM) or SJS/TEN (Table I). The strictly proximal distribution, large size of each primary lesion (2.5-4 cm) distributed over a background of diffuse postinflammatory hyperpigmentation, and rim of erythema are clinical evidence supportive of GBFDE rather than EM. Additionally, the patient only had involvement of one mucosal surface, whereas patients with SJS/TEN typically have more extensive mucosal involvement of 2 or more mucosal sites. Furthermore, the presence of eosinophils on histopathology are suggestive of a drug-induced process such as FDE rather than EM. The delayed onset of her eruption may have been related to intramuscular delivery of the offending drug.

**Table I:** Clinical factors that favor GBFDE over EM or SJS/TEN

	Primary morphology	Distribution	Body surface area involvement	Histopathologic features	Features of our patient
Fixed drug eruption	Well-demarcated red to brown patches or edematous plaques ± bullae with PIH	Any body site, but predilection for anogenital region, lips	Variable	Vacuolar interface dermatitis with lymphoeosinophilic infiltrate and pigment incontinence, ± dyskeratotic keratinocytes	Well-demarcated red-brown patches with overlying bullae and in a background of diffuse PIH; eosinophilic infiltrate on histopathology
EM	3-zone target lesions or atypical papular targets	Acral, initially on extremities, ± 1 mucosal surface	<10%	Lymphocytic vacuolar interface dermatitis, dyskeratosis throughout all layers of epidermis	Proximal distribution rather than acral; no typical targets, and most lesions were macular rather than papular atypical targets
SJS	Atypical macular targets with bullae formation	Trunk and proximal extremities, palms and soles; ≥2 mucosal surfaces	<10%	Confluent epidermal necrosis, minimal inflammatory infiltrate	Slowly progressive course; only 1 mucosal surface Histopathology found epidermal necrosis but with eosinophilic infiltrate body surface area <30%; involvement of only 1 mucosal surface
TEN	Atypical macular targets with bullae formation	Trunk and proximal extremities, palms and soles; ≥2 mucosal surfaces	>30%	Full-thickness epidermal necrosis, minimal inflammatory infiltrate	

*PIH*, Postinflammatory hyperpigmentation.

FDE is typically a benign and self-limiting reaction, resolving in days to weeks with topical therapy; however, GBFDE often requires more extensive treatment, with reports controlling for body surface involvement suggesting comparable mortality to SJS/TEN.^2^ Current evidence-based standards of care for management are lacking; however, some successful treatments of GBFDE include immunomodulatory agents such as cyclosporine and prednisone.^5^, ^6^ In our patient, progression despite initiation of oral prednisone warranted transfer to the burn intensive care unit where aggressive support prevented life-threatening fluid losses, electrolyte imbalances, and secondary infections. Further research into the pathophysiology and treatment are needed to reduce the morbidity and mortality of this condition.
